# Tobramycin Reduces Pulmonary Toxicity of Polymyxin B via Inhibiting the Megalin-Mediated Drug Uptake in the Human Lung Epithelial Cells

**DOI:** 10.3390/pharmaceutics16030389

**Published:** 2024-03-12

**Authors:** Maizbha Uddin Ahmed, Jian Li, Qi (Tony) Zhou

**Affiliations:** 1 Department of Industrial and Molecular Pharmaceutics, College of Pharmacy, Purdue University, 575 Stadium Mall Drive, West Lafayette, IN 47907, USA; 2 Monash Biomedicine Discovery Institute, Infection Program and Department of Microbiology, Monash University, Clayton, VIC 3800, Australia

**Keywords:** polymyxin, tobramycin, human lung epithelial cell, pulmonary toxicity, drug transporter, megalin

## Abstract

Accumulation of polymyxins in the lung epithelial cells can lead to increased mitochondrial oxidative stress and pulmonary toxicity. Aminoglycosides and polymyxins are used, via intravenous and pulmonary delivery, against multidrug-resistant Gram-negative pathogens. Our recent in vitro and animal studies demonstrated that the co-administration of polymyxins with aminoglycosides decreases polymyxin-induced pulmonary toxicity. The aim of this study was to investigate the in vitro transport and uptake of polymyxin B and tobramycin in human lung epithelial Calu-3 cells and the mechanism of reduced pulmonary toxicity resulting from this combination. Transport, intracellular localization, and accumulation of polymyxin B and tobramycin were investigated using doses of 30 mg/L polymyxin B, 70 mg/L tobramycin, and the combination of both. Adding tobramycin significantly (*p* < 0.05) decreased the polymyxin B-induced cytotoxicity in Calu-3 cells. The combination treatment significantly reduced the transport and uptake of polymyxin B and tobramycin in Calu-3 cells, compared to each drug alone, which supported the reduced pulmonary toxicity. We hypothesized that cellular uptake of polymyxin B and tobramycin shared a common transporter, megalin. We further investigated the megalin expression of Calu-3 cells using confocal microscopy and evaluated megalin activity using a megalin substrate, FITC-BSA, and a megalin inhibitor, sodium maleate. Both polymyxin B and tobramycin significantly inhibited FITC-BSA uptake by Calu-3 cells in a concentration-dependent manner. Sodium maleate substantially inhibited polymyxin B and tobramycin transport and cellular accumulation in the Calu-3 cell monolayer. Our study demonstrated that the significantly reduced uptake of polymyxin B and tobramycin in Calu-3 cells is attributed to the mechanism of action that determines that polymyxin B and tobramycin share a common transporter, megalin.

## 1. Introduction

Polymyxins (i.e., polymyxin B and colistin) are often the only available therapeutic option for Gram-negative ‘superbugs’ [1,2]. Unfortunately, after intravenous administration of polymyxins (as colistin methanesulfonate [CMS] or polymyxin B), very limited drug exposure is achieved in the lungs, which leads to poor efficacy in the treatment of lung infections [1,3,4]. Simply increasing the intravenous dose of polymyxins is not feasible due to the dose-limiting nephrotoxicity [2,3]. Several clinical studies have reported satisfactory clinical efficacy of polymyxin inhalation therapy for lung infections in patients with bronchiectasis, cystic fibrosis (CF), and pneumonia [5,6,7,8,9], even though current inhaled polymyxin therapies have never been subjected to systematic PK/PD/TD optimization [10].

Our earlier work revealed that polymyxin-induced apoptosis in human lung epithelial A549 cells is primarily due to the mitochondrial, death receptor, and endoplasmic reticulum pathways [11,12]. Moreover, we integrated correlative synchrotron-based X-ray fluorescence microscopy (XFM) and confocal fluorescence microscopy and quantitatively mapped, for the first time, the substantial accumulation of polymyxins in single A549 cells [13]. Using metabolomics, we revealed that polymyxins significantly perturbed mitochondrial β-oxidation, membrane lipid biogenesis, and cellular redox balance [14]. Therefore, there is an urgency to develop novel strategies for attenuation of polymyxin-induced pulmonary toxicity, thereby improving the efficacy of inhaled polymyxin therapy and minimizing the development of antimicrobial resistance [15].

Several studies have shown that the combination of aminoglycosides and polymyxins is superior to monotherapy options in the treatment of lung infections caused by *Pseudomonas aeruginosa* [16,17,18,19]. We discovered that the combination of polymyxins with aminoglycosides could significantly decrease polymyxin-induced pulmonary toxicity both in vitro and in vivo [20]. However, the mechanism of such effects is unknown. Megalin is a large, multiligand, endocytic-membrane glycoprotein expressed in the epithelial cells of multiple organs, including the intestines, kidneys, lungs, and brain [21]. Megalin is responsible for endocytic uptake of many biological ligands as well as drugs such as vancomycin, cisplatin, aminoglycosides, and polymyxins in epithelia [21,22,23,24]. Megalin plays a significant role in the renal accumulation of aminoglycosides [23] and polymyxins [22,24,25]. Although megalin is expressed in the lungs, the uptake of aminoglycosides and polymyxin B by megalin has not been examined in lung epithelial cells. The present study aims to investigate the role of megalin transport in reduced pulmonary toxicity observed when the combination of tobramycin and polymyxin B is administered (versus monotherapy).

## 2. Materials and Methods

### 2.1. Materials

Polymyxin B (sulfate) and tobramycin (sulfate) were purchased from Betapharma Co., Ltd. (Wujiang, China). LDH assay kits, 10% formalin, DAPI, BCA protein assay kit, TritonX-100, and RIPA buffer were obtained from Thermo Fisher scientific Inc. (Waltham, MA, USA). The fetal bovine serum (FBS), Dulbecco’s Modified Eagle Medium (DMEM), Hanks’ Balanced Salt Solution (HBSS), mouse anti-polymyxin B IgM, and goat anti-mouse IgM antibody conjugated with Alexa Fluor™ 647 were purchased from Life Technologies Corporation (Eugene, OR, USA). Paraformaldehyde (PFA) was purchased from Biotium, Inc. (Fremont, CA, USA). Transwell^®^ (6.5 mm), and 0.4 µm Pore Polyester Membrane Insert was purchased from Corning (Glendale, AZ, USA).

### 2.2. Cell Culture

Human lung epithelial Calu-3 cells were grown in a 6.5 mm cell culture insert in a trans-well plate for 3 days using both apical and basal media. The apical media was withdrawn to let the cells grow in an air liquid interface (ALI) [26]. The system was maintained for 14 days, with the basal media changed every other day. The culture medium was DMEM/F-12 supplemented with 10% (*v*/*v*, FBS) and 1% (*v*/*v*) nonessential amino acid, and penicillin and streptomycin (100 units/mL of penicillin and 100 mg/L of streptomycin). After 14 days of differentiation at the ALI condition, the trans-epithelial electrical resistance (TEER) value was measured to investigate the integrity of the formed Calu-3 monolayer.

### 2.3. Cytotoxicity Assessment

Assessment of cytotoxicity was performed using aqueous solutions of polymyxin B, tobramycin, and the combination of both in human lung epithelial Calu-3 cells. This cytotoxicity assessment aimed to determine safe drug concentrations for the transport and imaging experiments. Cells were seeded in 96-well flat bottom plate (Corning, NY, USA) at a density of 2 × 10^5^ cells/mL. A wide concentration range was chosen, considering a molar ratio of 1:5 polymyxin B to tobramycin based on the literature [20]. Lactate dehydrogenase (LDH) assay was used for the measurement of cytotoxicity. Vehicle-treated cells were considered as the negative control, while 10× lysis buffer treated cells were employed as the positive control. For the imaging study, Calu-3 cells were cultured in a 35 mm glass bottom culture dish at a density of 2 × 10^5^ cells/mL and were treated with a vehicle, tobramycin 700 mg/L, polymyxin B 300 mg/L, and the combination. After treatment, cells were washed with PBS and stained with PI (100 ng/mL) and DAPI (1 mg/L) for 30 min in dark. Cells were washed again with PBS and mounted for imaging with Calu-3 cell culture media.

### 2.4. Transport Study

The transport study was conducted in the Calu-3 cell monolayer according to our previously published ALI culture protocol [26]. TEER values were measured before and after the transport study to ensure the cell monolayer integrity was maintained. As the transport process was done in HBSS buffer, the drug solution was also prepared in HBSS buffer. The cell monolayer inserts were incubated with HBSS buffer for 30 min to equilibrate. The following drug solutions were used in the transport process: polymyxin B 30 mg/L, tobramycin 70 mg/L, and the combination of polymyxin B 30 mg/L and tobramycin 70 mg/L. An aliquot of 200 µL of drug solution was added to the apical chamber and 600 µL of HBSS buffer was added to the basal chamber. The samples were collected from the basal chamber every hour for a total of 4 h and the basal chamber was refilled with fresh HBSS buffer. Transported drug from the basal chamber, remaining drug from the apical chamber, and drug accumulated in the cell monolayer were extracted and analyzed using a reversed-phase LC/MS system. Drug extraction and LC/MS analysis was performed according to a previously published protocol [26,27,28].

### 2.5. LC-MS Analysis

For the transported samples, all buffer in the basal chamber was collected to a centrifuge tube. Colistin (1 mg/L) was added as the internal standard, and formic acid (0.1%, *v*/*v*) was added to each sample. For drug analysis in the cell, the insert was cut using a sharp scalpel. The cells in the membrane were lysed overnight with RIPA buffer and 200 µL of each sample was used to extract the drug using a protein precipitation method. Acetonitrile (400 µL) was added to the cell lysate and vortexed well to precipitate all the protein. Internal standard and formic acid were added to the samples as previously described, vortexed well, and centrifuged before transferring the supernatant into the injection vial [26].

The drugs were analyzed using an LC-MS/MS equipped with a C18 column (2.6 μm, 50 mm × 3 mm), a liquid chromatography system (Agilent 1200, Santa Clara, CA, USA), and a triple quadrupole mass spectrometer (Agilent 6460 QQQ, Santa Clara, CA, USA) with an electrospray ionization source [28]. The mobile phase was 0.1% formic acid solution in water (90%) and 0.1% formic acid solution in acetonitrile (10%) with a flow rate of 0.6 mL/min. A gradient elution procedure was used, and each component was measured via multiple reaction monitoring (MRM) in the positive ionization mode (Table 1) [29].

#### Cellular Accumulation and Distribution of Polymyxin B in the Calu-3 Cell Monolayer

Polymyxin B distribution in the Calu-3 cell monolayer was investigated using confocal laser scanning microscopy (CLSM) [26]. At the end of the transport process, the Calu-3 cell monolayer was prepared for imaging. The cell monolayer was washed with phosphate-buffered saline after treatment and fixed with 4% paraformaldehyde for 15 min. The cell monolayer was then permeabilized with 0.1% triton X-100 for 10 min and blocked with 3% goat serum in 0.1% triton X-100 for 2 h. To stain intracellular polymyxin B, the cell monolayer was then incubated overnight with mouse anti-polymyxin B antibody at 4 °C in the dark. After overnight incubation, the cell monolayer was incubated with goat anti-mouse IgG conjugated with Alexa Flour™ 647 for 2 h at room temperature. The nuclei were stained with Hoechst 33342.

For the drug accumulation study, Calu-3 cells were cultured in a glass bottom 35 mm dish (Ashland, MA, USA) and were incubated with 30 mg/L polymyxin B in the absence and presence of 70 mg/L tobramycin for 4 h. Cells were prepared for imaging using the same protocol as followed for the cell monolayer. Images were taken using Nikon confocal microscopy (Nikon A1R, Nikon America Inc., Melville, NY, USA) with a 20× objective. For the drug distribution study, a multilayer Z-stake was set for imaging of the cell monolayer from the top to the bottom. A 3-D image was created from the obtained images of the Z-stack to determine the polymyxin B distribution throughout the cells [26]. The settings for camera aperture, image capture and gain parameters, and laser poser were adjusted with untreated control and maintained throughout the study. For the drug accumulation study, the background subtracted integrated density of Alexa Flour 647 (for polymyxin B) was calculated and data were presented as mean polymyxin B intensity per cell. Cell numbers per imaging field were counted using the Hoechst 33342 intensity and ImageJ “cell counter” Macro (ImageJ version 2.9).

### 2.6. Distribution of Megalin Transporter in Calu-3 Cells

Calu-3 cells were grown in eight-well chamber slides (Catalogue 80841, Ibidi, Fitchburg, WI, USA) at a density of 2 × 10^5^ cell/mL and incubated overnight at 37 °C and 5% CO_2_. Cells were then washed with PBS and fixed with 4% paraformaldehyde, permeabilized with 0.1% triton X-100, and blocked with 3% goat serum in 0.1% triton X-100. Cells were incubated overnight at 4 °C in the dark with megalin/LRP2 Antibody conjugated with Alexa Fluor^®^ 488 (Catalogue sc-515750 AF488, Santa Cruz Biotechnology, Dallas, TX, USA). Cells were washed and incubated with Hoechst 33342 (Catalogue 62249, 1 mg/L, Thermofisher Scientific, Waltham, MA, USA) for nucleus staining and cell mask deep red (Catalogue C10046, 1X, Thermofisher Scientific, USA) for cell membrane staining. Cells were finally washed and covered with Prolong gold antifade mounting reagent (Catalogue P36934, Thermofisher Scientific, USA) and mounted on a 60× oil immersion objective for imaging. Images were taken with a Nikon confocal microscope (Nikon Eclipse T1 confocal, Nikon, Melville, NY, USA). Images were processed with Nikon (NIS-Elements) and Image J software (version 1.54).

### 2.7. Evaluation of Megalin Substrate Uptake by Calu-3 Cells in Presence of Polymyxin B and Tobramycin

As albumin is an endogenous substrate of megalin [30], cellular distribution of fluorescein-labeled BSA (FITC-BSA) was evaluated in the presence of polymyxin B and tobramycin to investigate the functional activity of megalin in Calu-3 cells. Calu-3 cells were grown in an eight-well chamber slide at a density of 2 × 10^5^ cell/mL in 10% FBS supplemented DMEM/F-12 cell culture media. After overnight incubation at 37 °C and 5% CO_2_, cells were washed to remove old media and were incubated for 4 h with 100 μg/mL of FITC-BSA in fresh cell culture media and in the presence and absence of polymyxin B (30 and 60 mg/L) and tobramycin (70 and 140 mg/L). After 4 h, cells were washed and stained with Hoechst for nucleus staining and cell mask deep red for cell membrane staining. Cells were finally washed and covered with Prolong gold antifade mounting reagent and mounted on a 60× oil immersion objective for imaging. Images were taken with a Nikon Eclipse T1 confocal microscope. Images were processed with Nikon (NIS-Elements) and Image J (version 1.54) software.

### 2.8. Evaluation of Polymyxin B and Tobramycin Transport Behavior in Calu-3 Cells in Presence of Megalin Inhibitor

The transport behavior of polymyxin B and tobramycin in the Calu-3 cell monolayer were also evaluated in the presence of 8 mg/mL of sodium maleate, an agent known to inhibit megalin receptors via shedding of the megalin protein [23]. Calu-3 cell monolayer in trans-well inserts was formed following our previously published ALI culture protocol [26]. Cell monolayers were pre-incubated with 8 mg/mL of sodium maleate for 4 h and were subsequently incubated with polymyxin B and tobramycin for 2 h. Samples were collected from the apical and basal chambers of the ALI culture system, analyzed using an LC-MS/MS system, and the drug content was calculated. The cell monolayer was then lysed with lysis buffer (RIPA buffer, Catalogue 89900, Thermofisher Scientific, Waltham, MA, USA) and cellular drug content was measured. Cell lysate (25 µL) from each sample was used for protein measurement. The amount of drug accumulated in the cell was normalized according to the amount of protein present in the samples. Polymyxin B and tobramycin concentrations in the samples, in the presence and absence of the megalin inhibitor sodium maleate, were plotted and statistical significance was calculated using Graph Pad Prism (version 9).

## 3. Results

### 3.1. Effect of Tobramycin on the Polymyxin-Induced Cytotoxicity in Calu-3 Cells

**Figure 1:**
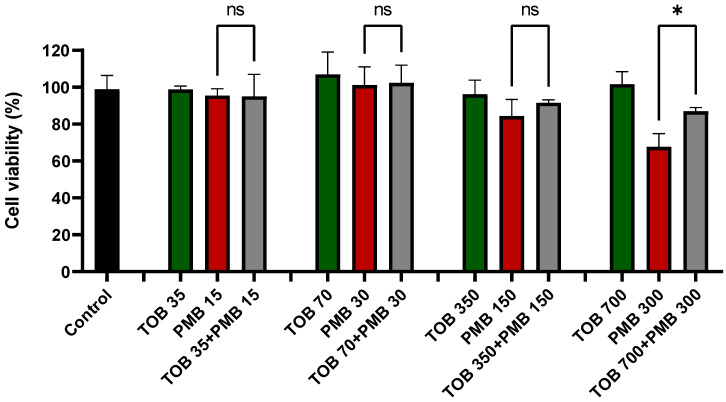
Cell viability in human lung epithelial Calu-3 cells. PMB: Polymyxin B; TOB: Tobramycin. Each number in the x axis indicates concentrations in mg/L. Combinations have a polymyxin and tobramycin molar ratio of 1:5 or equivalent mass ratio of 3:7. Data are presented as mean ± SD (n = 3). * *p* < 0.05. ns = not significant.

**Figure 2:**
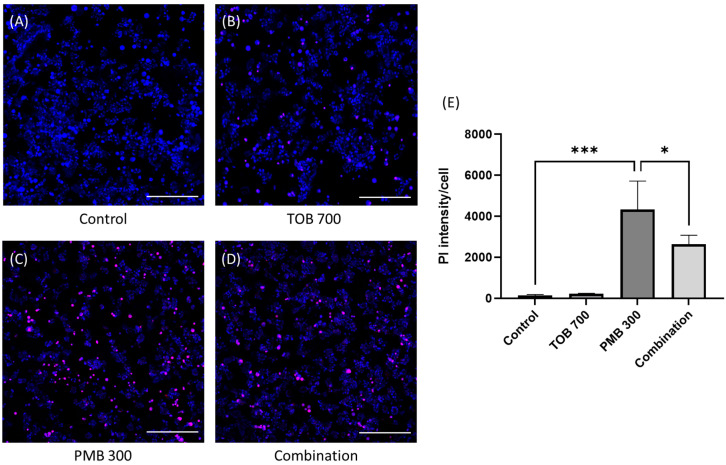
Fluorescence microscopic investigation of control (**A**), tobramycin (**B**), polymyxin B (**C**), and combination (**D**) toxicity in human lung epithelial Calu-3 cells. PMB: Polymyxin B; TOB: Tobramycin. Each number in the x-axis indicates concentrations in mg/L. Combinations have a polymyxin B and tobramycin molar ratio of 1:5 or equivalent mass ratio of 3:7. Data are presented (**E**) as mean ± SD (n = 3). Scale bar = 100 µm. * and *** indicate statistical significance of *p* < 0.05 and *p* < 0.001, respectively. Blue color indicates nucleus of the cell and red color indicates the PI positive cells.

Tobramycin alone and in combination with polymyxin B treatments did not result in any reduction in the viability of lung epithelial Calu-3 cells compared to the control (Figure 1). In contrast, polymyxin B caused significant cell death at 300 mg/L as compared to the control. After 24 h of treatment with polymyxin B alone at 300 mg/L, the cell viability was 67.7 ± 5.8%, which was significantly lower than the combination (87.0 ± 1.6%, *p* < 0.0001). Based on cell viability data, 30 mg/L of polymyxin B alone, 70 mg/L of tobramycin alone, and their combination were chosen for the transport study as these concentrations were safe for the Calu-3 cell monolayer (Figure 1). Imaging analysis showed that PI intensity significantly increased (4334.0 ± 1131.6, *p* < 0.001 compared to the control samples) in the polymyxin B (300 mg/L) treated lung epithelial Calu-3 cells (Figure 2C,E), which was substantially reduced (2644.8 ± 351.6; *p* < 0.05 compared to polymyxin B 300 mg/L treated samples) by the concurrent use of tobramycin 70 mg/L (Figure 2D,E). The saline control (Figure 2A,E) and tobramycin (Figure 2B,E) showed minimum toxicity.

### 3.2. Transport of Polymyxin and Tobramycin in the Air Liquid Culture Model of Calu-3

**Figure 3:**
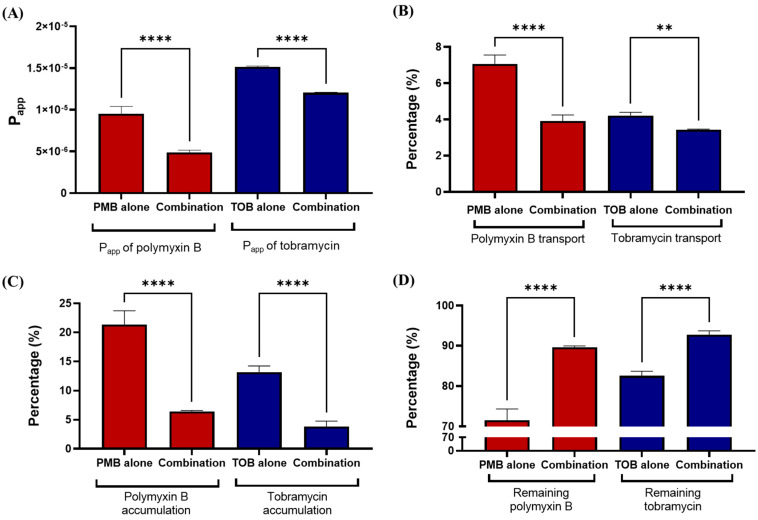
Polymyxin B and tobramycin transport in Calu-3 cells: (**A**) Apparent permeability coefficient (P_app_) of drug transport. (**B**) Transport of polymyxin B and tobramycin from apical chamber to basal medium. (**C**) Cellular accumulation of polymyxin B and tobramycin. (**D**) Remaining polymyxin B and tobramycin in the apical chamber of the trans-well insert after 4 h of transport study. ** *p* < 0.01 and **** *p* < 0.0001 represents the statistical significance. PMB: polymyxin B; TOB: tobramycin.

Transport of polymyxin B and tobramycin in Calu-3 cells was significantly affected by combination of the compounds (Figure 3). The transport rate, calculated by apparent permeability coefficient (P_app_) of both polymyxin B and tobramycin, decreased significantly (*p <* 0.0001) when used in combinations, as compared to polymyxin B and tobramycin alone (Figure 3A). Polymyxin B transport in polymyxin B alone was 7.1 ± 0.4%, which was reduced to 3.9 ± 0.3% (*p* < 0.0001) by the combination (Figure 3B). Both polymyxin B and tobramycin accumulations in Calu-3 cell monolayer were significantly decreased by the combination as compared to either drug alone (Figure 3C). Polymyxin B accumulation substantially decreased from 21.4 ± 2.0% for polymyxin B alone to 6.4 ± 0.1% (*p* < 0.0001) for the combination, while the accumulation of tobramycin was 13.2 ± 4.3% for tobramycin alone at 4 h but 3.8 ± 0.8% (*p* < 0.0001) for the combination treatment. Significantly higher amounts of polymyxin B and tobramycin remained in the apical chamber when they were administered as part of the combination as compared to polymyxin B or tobramycin alone (Figure 3D).

### 3.3. Cellular Uptake and Distribution of Polymyxin B in Calu-3 Cell Monolayer by Confocal Microscopy

**Figure 4:**
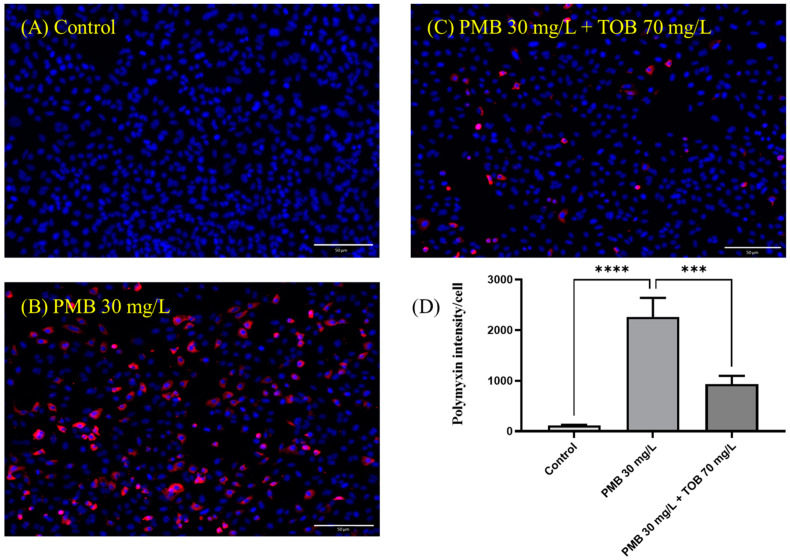
Polymyxin B accumulation in Calu-3 cell monolayer after 4 h of treatment. (**A**) Saline treated control, (**B**) Polymyxin B 30 mg/L treated cells, (**C**) Combination of polymyxin B 30 mg/L and tobramycin 70 mg/L treated cells and (**D**) Polymyxin B intensity plotted against the treatment. Images presented in the figure are representative of three or more images taken from different areas of the culture dish. Polymyxin B intensity (red) was calculated as per cell and plotted against control. The nucleus is in blue. Data are presented as mean ± SD, scale bar = 50 µm. *** *p* < 0.001 and **** *p* < 0.0001 value was calculated for the combination treatment as compared to the polymyxin B alone treatment.

**Figure 5:**
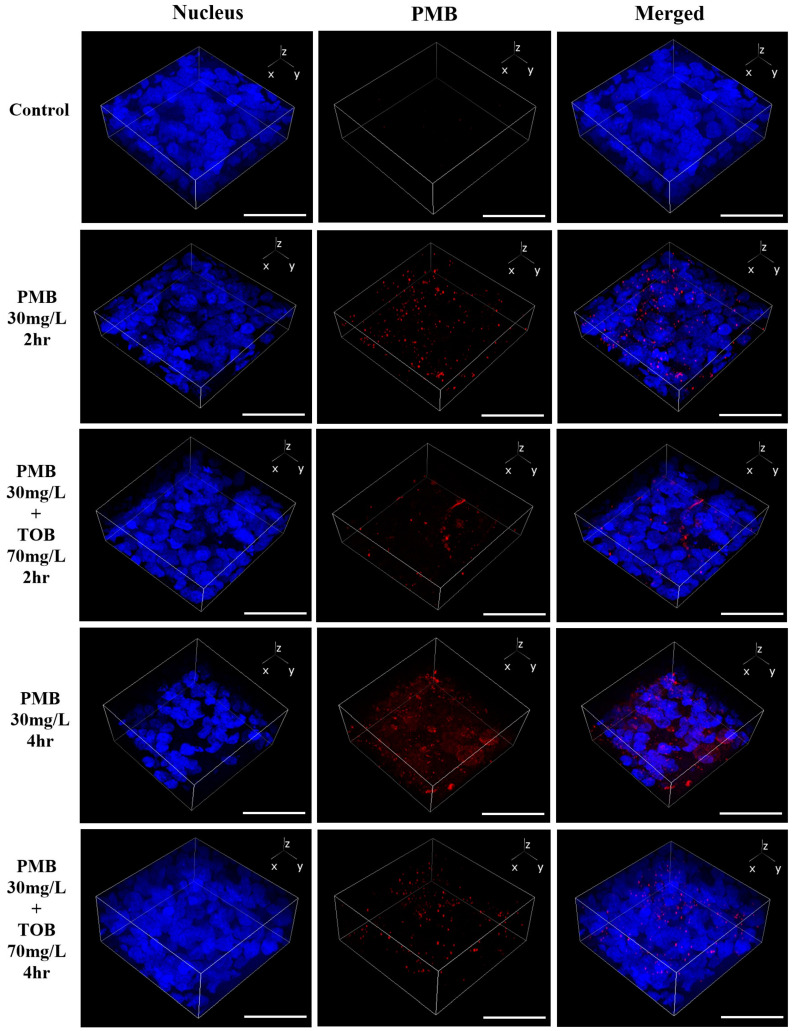
Accumulation of polymyxin B (in red) in Calu-3 cell monolayer. Background noise for the red intensity was normalized with the control cell monolayer (no treatment). Nucleus is in blue, and the scale bar is 50 µm.

Cellular uptake of polymyxin B in Calu-3 cell monolayer was calculated as polymyxin B intensity per cell. It is evident that polymyxin B uptake (red intensity) in Calu-3 cell monolayer was substantially decreased by the combination as compared to polymyxin B (30 mg/L) alone (Figure 4D). After 4 h of treatment, polymyxin B intensity per cell was substantially higher (2259.3 ± 309.4, *p* = 0.0001) in the polymyxin B treated cells (Figure 4B,D) compared to the saline controls (113.0 ± 13.6, Figure 4A,D), which was substantially reduced to 932.3 ± 136.0 (*p* < 0.001) by the combination (Figure 4C,D). Polymyxin B was tagged with red fluorophore (Alexa 647) and imaged with confocal microscopy. A 3-D view of the Calu-3 cell monolayer was created from the images obtained *via* Z-stacking of monolayer from top to bottom. It is evident that the accumulation of polymyxin B in the Calu-3 cell monolayer was substantially reduced by the combination at both 2 h and 4 h (Figure 5).

### 3.4. Megalin Distribution and Activity in Calu-3 Cells

**Figure 6:**
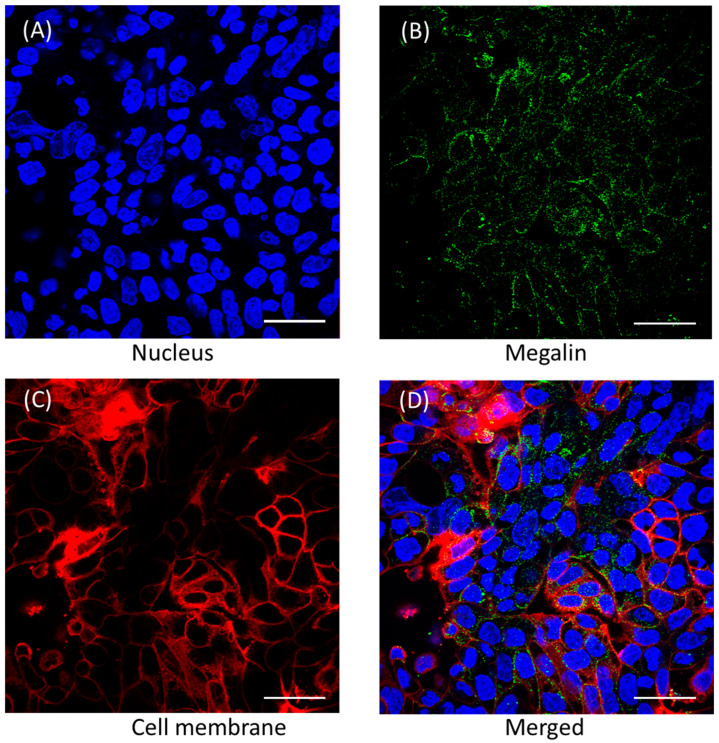
Distribution of megalin in Calu-3 cells. (**A**) Nucleus, (**B**) megalin, (**C**) cell membrane, and (**D**) merged. Scale bar = 50 µm.

**Figure 7:**
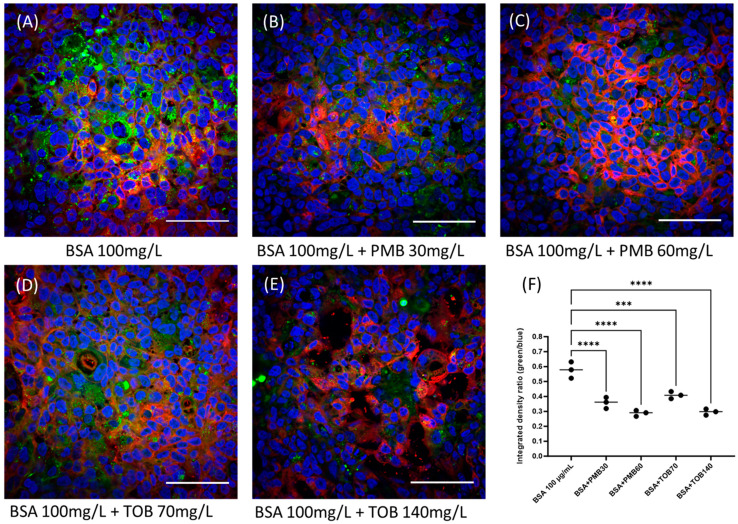
Effect of polymyxin B and tobramycin on the cellular distribution of BSA (FITC-BSA). Scale bar = 100 µm. Statistical significance *** *p* < 0.001, **** *p* < 0.0001. Nucleus is in blue, BSA (FITC-BSA) is in green, and cell membrane tracker is in red.

Confocal imaging analysis of Calu-3 cells grown on eight-well chamber slides demonstrated the presence of megalin (Figure 6). In order to assess megalin activity in Calu-3 cells, cellular distribution of FITC-BSA, a megalin substrate, was evaluated in the presence and absence of polymyxin B and tobramycin using confocal microscopy (Figure 7). Both polymyxin B and tobramycin prevented the cellular uptake of FITC-BSA in a concentration-dependent manner. The fluorescence intensity of FITC-BSA was normalized throughout all the samples by the ratio of FITC-BSA to nucleus integrated density (Figure 7). The ratio of 100 mg/L FITC-BSA only was 0.58 ± 0.04, which was reduced significantly to 0.36 ± 0.03 (**** *p* < 0.0001) and 0.29 ± 0.02 (**** *p* < 0.0001) by 30 mg/L and 60 mg/L of polymyxin B, respectively. The ratio was substantially reduced to 0.41 ± 0.02 (*** *p* < 0.001) and 0.30 ± 0.02 (**** *p* < 0.0001) by 70 mg/L and 140 mg/L of tobramycin, respectively.

**Figure 8:**
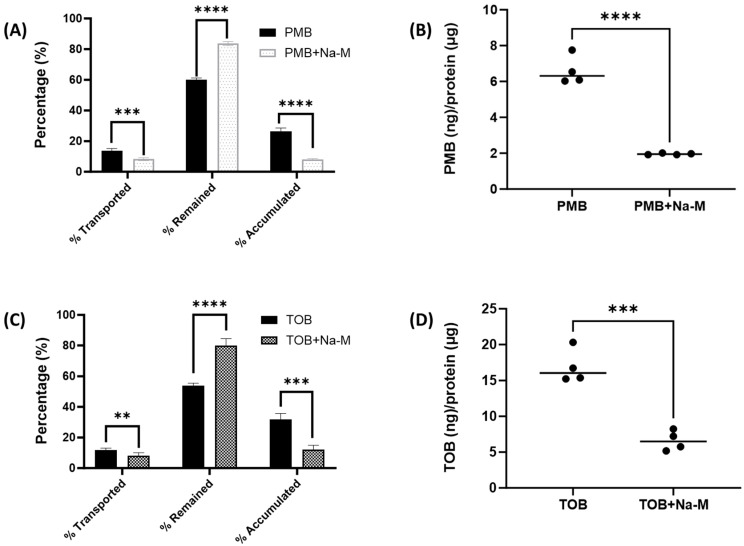
Effect of sodium maleate on the transport behavior of polymyxin B and tobramycin in Calu-3 cell monolayer. (**A**) Transport summary of polymyxin B in presence and absence of sodium maleate. (**B**) Effect of Sodium maleate on cellular accumulation of polymyxin B. (**C**) Transport summary of tobramycin in presence and absence of sodium maleate. (**D**) Effect of sodium maleate on cellular accumulation of tobramycin. Statistical significance: ** *p* < 0.01, *** *p* < 0.001, and **** *p* < 0.0001.

To further assess megalin activity, we investigated polymyxin B and tobramycin transport behavior in Calu-3 cells in the presence of sodium maleate, a compound known to inhibit megalin receptors through shedding megalin protein. Our data showed that sodium maleate significantly reduced the transport of polymyxin B and tobramycin from the apical chamber to the basal chamber of the trans-well insert in Calu-3 cells (Figure 8). For instance, 13.5 ± 1.6% of polymyxin B was transported from the apical chamber to the basal chamber in the polymyxin B alone sample, which was significantly reduced to 8.2 ± 0.9% (*** *p* < 0.001) in the presence of sodium maleate. Similarly, cellular accumulation of polymyxin B dropped substantially to 8.1 ± 0.4% from 26.5 ± 2.1% (**** *p* < 0.0001), whereas remaining polymyxin B in the apical chamber increased from 59.9 ± 1.2% to 83.7 ± 1.3% (**** *p* < 0.0001) due to the effect of sodium maleate. Intracellular accumulation of polymyxin significantly dropped from 6.0 ± 0.8 (ng/µg of protein) to 1.9 ± 0.04 (ng/µg of protein) with the presence of sodium maleate (**** *p* < 0.0001). On the other hand, 11.9 ± 1.3% of tobramycin was transported from the apical chamber to the basal chamber in the tobramycin alone sample, which was significantly reduced to 8.0 ± 2.2% (** *p* < 0.01) in the presence of sodium maleate. Similarly, cellular accumulation of tobramycin reduced substantially to 12.2 ± 2.8% from 31.7 ± 2.1% (*** *p* < 0.001), whereas remaining tobramycin in the apical chamber increased from 53.9 ± 1.7% to 79.9 ± 4.7% (**** *p* < 0.0001) with the presence of sodium maleate. Intracellular accumulation of tobramycin significantly reduced from 16.9 ± 2.4 (ng/µg of protein) to 6.6 ± 1.4 (ng/µg of protein) under the influence of sodium maleate (**** *p* < 0.0001).

## 4. Discussion

Polymyxin inhalation therapy offers a targeted approach for treating respiratory tract infections caused by multidrug-resistant Gram-negative bacteria [31]. Inhalation therapy allows for direct delivery of polymyxins to the lungs, achieving high concentrations at the site of infection while minimizing systemic exposure and potential side effects [32]. Inhaled polymyxins may cause pulmonary toxicity due to a lack of dose optimization. In human lung epithelial cells, polymyxins cause concentration- and time-dependent toxicity [12,33]. Pulmonary epithelial cells are rich in mitochondria and mitochondrial dysfunction plays a significant role in lung pathogenesis when exposed to toxicants [33]. Substantial accumulation of polymyxins within mitochondria results in mitochondrial damage in lung epithelial A549 cells [11]. Our work discovered that aminoglycosides can attenuate polymyxin-induced respiratory toxicity [20].

Our data here exhibited that transport of both polymyxin B and tobramycin across the Calu-3 cell monolayer was significantly reduced when they were administered together as compared to each drug alone (Figure 3). Our study showed that co-administration of tobramycin reduced cellular accumulation (Figure 4 and Figure 5) of polymyxin B and therefore increased the viability of human lung epithelial Calu-3 cells (Figure 1). Both compounds affected the transport behavior of the other compound in Calu-3 cells (Figure 3). To elucidate the mechanism of the effect of tobramycin on the uptake and transport of polymyxins in human lung epithelial Calu-3 cells, we investigated the role of megalin. Lung tissues are rich in megalin. Different studies have demonstrated that cellular uptake of tobramycin and polymyxin is mediated by megalin in the renal tubular epithelium, alveolar epithelium, and gastrointestinal epithelium [23,24,34,35,36,37]. Suzuki et al. reported that colistin acts as a megalin ligand and that the presence of megalin plays a key role in the accumulation of colistin in kidney tubular cells and nephrotoxicity [24]. Megalin also plays a significant role in aminoglycoside-induced nephrotoxicity [23,37]. It is highly likely that tobramycin reduced polymyxin uptake in the lung epithelial Calu-3 cells (Figure 4 and Figure 5) via inhibition of megalin receptors, which resulted in attenuated cell death (Figure 1 and Figure 2).

This study is the first to evaluate the expression of megalin in lung epithelial Calu-3 cells using confocal microscopy (Figure 6) and examine the involvement of megalin in the uptake of polymyxin B and tobramycin (Figure 7). Using a fluorescent protein FITC-BSA, a megalin substrate, our data showed that treating the cells with polymyxin B or tobramycin reduced the cellular uptake of FITC-BSA [38]. Collectively, our data indicated that the uptake of FITC-BSA is a megalin transporter-mediated process, as both polymyxin B and tobramycin inhibited FITC-BSA uptake in lung epithelial cells in a concentration dependent manner [23,38]. Our results also demonstrated that sodium maleate, a megalin receptor inhibitor, decreased both polymyxin B and tobramycin transport and cellular accumulation in Calu-3 cells. Maleate inhibited ~60% of the uptake of both polymyxin B and tobramycin (Figure 8). This suggests that polymyxin B and tobramycin have a similar inhibitory effect on megalin. Our findings are consistent with earlier reports by Suzuki et al. [24] that showed a significant reduction in colistin accumulation in megalin-shedding rats, and those by Nagai et al. [23] that showed that 400 mg/kg of sodium maleate administration decreased renal cortex accumulation of amikacin by 50% within 3 h after injection. Megalin knock-out mice also demonstrated reduced renal accumulation of aminoglycosides [37]. Tobramycin was reported to be bound to megalin (K_d_ = 2.4 mM) and cubulin (K_d_ = 2.4 mM) [39]. We believe tobramycin inhibited the transport and accumulation of polymyxin in Calu-3 cells and reduced polymyxin-induced pulmonary toxicity through its interaction with megalin.

## 5. Conclusions

This study is the first to unveil the mechanism of reduced polymyxin-induced pulmonary toxicity *via* co-administration of aminoglycosides. The combination of polymyxin B and tobramycin reduced transport of both drugs from the apical chamber to the basal media and decreased intracellular accumulation of both drugs in human lung epithelial Calu-3 cells. The expression of megalin and its role in polymyxin and tobramycin transport in lung epithelial cells has been investigated for the first time in this study. Such significantly decreased uptake of both drugs in human lung epithelial cells contributed to reduced pulmonary toxicity caused by accumulation of polymyxins. Our results support that the combination therapy of polymyxins and aminoglycosides can be a promising treatment option for lung infections associated with MDR Gram-negative bacteria. 

## Figures and Tables

**Table 1:** MS conditions for the detection of polymyxin B and tobramycin.

Compound Name	Precursor Ion (*m*/*z*)	Product Ion (*m*/*z*)	Collision Energy (V)
Colistin A	585.3	241.1	15
Colistin B	578.3	227.0	15
Polymyxin B1	602.3	241.1	15
Polymyxin B2	595.3	227.1	15
Tobramycin	468.2	163.1	15

## Data Availability

Data are contained within the article.
